# Adaptations for finding irregularly shaped disease clusters

**DOI:** 10.1186/1476-072X-6-28

**Published:** 2007-07-05

**Authors:** Nikolaos Yiannakoulias, Rhonda J Rosychuk, John Hodgson

**Affiliations:** 1 School of Geography and Earth Sciences, McMaster University, Hamilton, Canada; 2 Department of Pediatrics, University of Alberta, Edmonton, Canada; 3 Department of Earth and Atmospheric Sciences, University of Alberta, Edmonton, Canada

## Abstract

**Background:**

Recent adaptations of the spatial scan approach to detecting disease clusters have addressed the problem of finding clusters that occur in non-compact and non-circular shapes – such as along roads or river networks. Some of these approaches may have difficulty defining cluster boundaries precisely, and tend to over-fit data with very irregular (and implausible) clusters shapes.

**Results & Discussion:**

We describe two simple adaptations to these approaches that can be used to improve the effectiveness of irregular disease cluster detection. The first adaptation penalizes very irregular cluster shapes based on a measure of connectivity (non-connectivity penalty). The second adaptation prevents searches from combining smaller clusters into large super-clusters (depth limit). We conduct experiments with simulated data in order to observe the performance of these adaptations on a number of synthetic cluster shapes.

**Conclusion:**

Our results suggest that the combination of these two adaptations may increase the ability of a cluster detection method to find irregular shapes without affecting its ability to find more regular (i.e., compact) shapes. The depth limit in particular is effective when it is deemed important to distinguish nearby clusters from each other. We suggest that these adaptations of adjacency-constrained spatial scans are particularly well suited to chronic disease and injury surveillance.

## Background

### Disease hot-spot detection

Cluster detection methods can be used to identify anomalous patterns of disease, and offer clues to the presence and location of emerging diseases and environmental hazards. However, these methods can face an important conceptual challenge if one adheres to traditional practices of hypothesis testing. It is difficult to formulate a single discrete null hypothesis about whether or not a cluster at a particular location is noteworthy unless we know the location of that cluster ahead of time. Since this is often the purpose of a cluster detection exercise, we are caught in a 'catch-22' – wanting to decide about the anomalousness of something that we have yet to find. One way this has been avoided is by stepping out of the traditional hypothesis testing paradigm – for example, by using the exploratory disease cluster detection tools [1,2]. These alternatives follow an inductive scientific model, and are well suited to exploring various patterns of events in space without a single explicit hypothesis test.

Turnbull et al. offer a disease cluster detection approach, the cluster evaluation permutation procedure (CEPP), which remains within the traditional hypothesis testing paradigm [3]. They conceive a method of geographic disease cluster detection that is focused on a most noteworthy cluster of disease events. In this form, the cluster detection process can be thought of as two distinct activities. First, search a system of disease data for a geographical subset that is more likely than any other (given the search criteria) to be an anomalous cluster. Second, test whether or not this cluster could have occurred by chance. In this form, a single hypothesis is being tested – is the cluster that is most likely to be anomalous actually anomalous? In their original work, the search proceeds by moving a circular window over the centroids of census areas. The window radius is fixed by a population threshold such that each window has approximately the same population. The window with the highest rate of disease is deemed the most-likely to be an anomalous cluster, and Monte Carlo methods are used to test a null hypothesis of constant risk.

This approach was generalized and more explicitly rationalized by Kulldorff, in the 'spatial scan' approach to disease cluster detection [4]. The spatial scan avoids the population size constraint of the CEPP by using a window of varying size. The potential cluster with the largest likelihood-ratio statistic is treated as the most-likely cluster to reject a null hypothesis of constant risk. If the test statistic is large enough to reject a null hypothesis, the cluster that caused the rejection remains anomalous (i.e., statistically significant) regardless of how the data outside the cluster are re-arranged. This is important since it links the approximate geographic location and size of a cluster to the rejection of the null-hypothesis. By restricting the search for most-likely clusters, this approach escapes the catch-22 noted above.

Recent adaptations of the spatial scan approach consider the problem of finding disease clusters that occur in non-compact and non-circular shapes – such as along roads or river networks. Some of these methods make use of adjacency characteristics rather than a pre-defined geometric or topologic structure to make the search for clusters efficient [5-7]. These new methods offer opportunities to better understand the precise geographic structure of clusters, as well as improving the power of detection when disease clusters occur in irregular shapes. However, they also confront a number of methodological challenges and performance issues [8,9]. As such, it is unclear if the benefits of being able to detect irregular clusters of disease (when they occur) offset the limitations associated with their use.

In this article we briefly review some recently developed adjacency-constrained alternatives to finding irregularly shaped clusters based on the test statistic associated with the spatial scan. We then describe two small, general adaptations that can be applied to adjacency-constrained cluster detection searches methods. These adaptations are designed to improve the accuracy of detection (reduce the locations falsely identified as part of a cluster), increase the ability of these methods to find disease clusters, and speed up the search process. Finally, these adaptations are tested on simulated data in order to observe how future implementations of adjacency-constrained spatial scan methods can bypass some of the existing challenges.

### General properties of adjacency-constrained spatial scan methods

**Figure 1:**
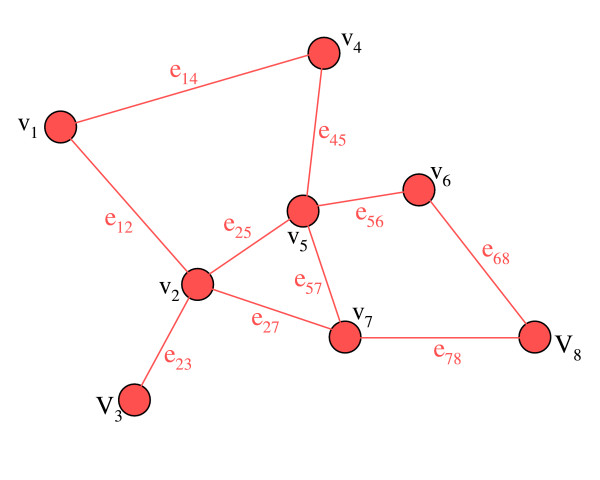
A system of polygonal areas conceived as a graph.

We define a study region that consists of a tessellation of *I *polygonal regions *v*_1_, *v*_2_,...,*v*_I_. When two regions, *v*_*i *_and *v*_*j *_meet a definition of adjacency, we treat them as neighbours. Using this convention, a tessellation of regions can be conceived as a graph of vertices and edges (Figure 1). Edges connect vertices in a way that reflects the topology of the polygons the vertices are meant to represent. We can assign a weight, *w*_*ij*_, to each edge *e*_*ij *_in the graph that characterizes the relationship between two connected nodes. These weights can represent geographic distance, or other attributes associated with the adjacent nodes. For a typical problem in disease cluster detection involving polygonal regions, there are a *y*_+ _disease cases and *d*_+ _population at risk in a study area. For each *v*_*i *_in V={vi}i=1I
 MathType@MTEF@5@5@+=feaafiart1ev1aaatCvAUfKttLearuWrP9MDH5MBPbIqV92AaeXatLxBI9gBaebbnrfifHhDYfgasaacH8akY=wiFfYdH8Gipec8Eeeu0xXdbba9frFj0=OqFfea0dXdd9vqai=hGuQ8kuc9pgc9s8qqaq=dirpe0xb9q8qiLsFr0=vr0=vr0dc8meaabaqaciaacaGaaeqabaqabeGadaaakeaacqWGwbGvcqGH9aqpcqGG7bWEcqWG2bGDdaWgaaWcbaGaemyAaKgabeaakiabc2ha9naaDaaaleaacqWGPbqAcqGH9aqpcqaIXaqmaeaacqWGjbqsaaaaaa@3986@, we have a number of disease cases *y*_*i *_and population *d*_*i *_(y+=∑i=1Iyi, d+=∑i=1Idi
 MathType@MTEF@5@5@+=feaafiart1ev1aaatCvAUfKttLearuWrP9MDH5MBPbIqV92AaeXatLxBI9gBaebbnrfifHhDYfgasaacH8akY=wiFfYdH8Gipec8Eeeu0xXdbba9frFj0=OqFfea0dXdd9vqai=hGuQ8kuc9pgc9s8qqaq=dirpe0xb9q8qiLsFr0=vr0=vr0dc8meaabaqaciaacaGaaeqabaqabeGadaaakeaacqWG5bqEdaWgaaWcbaGaey4kaScabeaakiabg2da9maaqadabaGaemyEaK3aaSbaaSqaaiabdMgaPbqabaaabaGaemyAaKMaeyypa0JaeGymaedabaGaemysaKeaniabggHiLdGccqGGSaalcqqGGaaicqWGKbazdaWgaaWcbaGaey4kaScabeaakiabg2da9maaqadabaGaemizaq2aaSbaaSqaaiabdMgaPbqabaaabaGaemyAaKMaeyypa0JaeGymaedabaGaemysaKeaniabggHiLdaaaa@4805@). We can estimate the rate of disease associated with each vertex with *y*_*i*_*/d*_*i*_.

Using this framework, the general adjacency constrained spatial scan approach can be conceived as a search to find a set of connected vertices from all possible sets of vertices that is more likely than any other set to be a cluster of disease. Each of these sets or 'potential clusters' is evaluated by calculating the likelihood ratio statistic (LR). Suppose *Z *is a set of connected vertices and *n*_*Z *_is the total number of cases within the vertices of *Z*. When searching for high clusters, the likelihood ratio statistic is

LR(Z)={(nZμZ)nZ(y+−nZy+−μZ)y+−nZnZ>μZ1Otherwise
 MathType@MTEF@5@5@+=feaafiart1ev1aaatCvAUfKttLearuWrP9MDH5MBPbIqV92AaeXatLxBI9gBaebbnrfifHhDYfgasaacH8akY=wiFfYdH8Gipec8Eeeu0xXdbba9frFj0=OqFfea0dXdd9vqai=hGuQ8kuc9pgc9s8qqaq=dirpe0xb9q8qiLsFr0=vr0=vr0dc8meaabaqaciaacaGaaeqabaqabeGadaaakeaacqWGmbatcqWGsbGucqGGOaakcqWGAbGwcqGGPaqkcqGH9aqpdaGabeqaauaabaqaciaaaeaadaqadaqaamaalaaabaGaemOBa42aaSbaaSqaaiabdQfaAbqabaaakeaaiiGacqWF8oqBdaWgaaWcbaGaemOwaOfabeaaaaaakiaawIcacaGLPaaadaahaaWcbeqaaiabd6gaUnaaBaaameaacqWGAbGwaeqaaaaakmaabmaabaWaaSaaaeaacqWG5bqEdaWgaaWcbaGaey4kaScabeaakiabgkHiTiabd6gaUnaaBaaaleaacqWGAbGwaeqaaaGcbaGaemyEaK3aaSbaaSqaaiabgUcaRaqabaGccqGHsislcqWF8oqBdaWgaaWcbaGaemOwaOfabeaaaaaakiaawIcacaGLPaaadaahaaWcbeqaaiabdMha5naaBaaameaacqGHRaWkaeqaaSGaeyOeI0IaemOBa42aaSbaaWqaaiabdQfaAbqabaaaaaGcbaGaemOBa42aaSbaaSqaaiabdQfaAbqabaGccqGH+aGpcqWF8oqBdaWgaaWcbaGaemOwaOfabeaaaOqaaiabigdaXaqaaiabd+eapjabdsha0jabdIgaOjabdwgaLjabdkhaYjabdEha3jabdMgaPjabdohaZjabdwgaLbaaaiaawUhaaaaa@6816@

where *μ*_*Z *_is the expected number of cases in the potential cluster under the null hypothesis [4]. The potential cluster of all possible clusters with the largest evaluation criteria is treated as the most-likely cluster, and is tested for significance. The evaluation process for the methods discussed below is approximately the same as used in the spatial scan; the methods differ from the original spatial scan with respect to how they search for clusters, and in that sense, most can be viewed as special cases of the original spatial scan approach.

### Examples of adjacency constrained spatial scans

There are a number of different ways to search sets of vertices for potential clusters of disease within this general approach. The size and complexity of a particular search approach is governed by the definition of adjacency used. At one extreme, vertices may be treated as connected only to themselves, and as such, the number of potential clusters available equals the total number of vertices. At another extreme, each vertex may be considered connected to all other vertices, which results in a total number of potential clusters equal to

∑k=1II!k!(I−k)!=2I−1.
 MathType@MTEF@5@5@+=feaafiart1ev1aaatCvAUfKttLearuWrP9MDH5MBPbIqV92AaeXatLxBI9gBaebbnrfifHhDYfgasaacH8akY=wiFfYdH8Gipec8Eeeu0xXdbba9frFj0=OqFfea0dXdd9vqai=hGuQ8kuc9pgc9s8qqaq=dirpe0xb9q8qiLsFr0=vr0=vr0dc8meaabaqaciaacaGaaeqabaqabeGadaaakeaadaaeWbqaamaalaaabaGaemysaKKaeiyiaecabaGaem4AaSMaeiyiaeIaeiikaGIaemysaKKaeyOeI0Iaem4AaSMaeiykaKIaeiyiaecaaaWcbaGaem4AaSMaeyypa0JaeGymaedabaGaemysaKeaniabggHiLdGccqGH9aqpcqaIYaGmdaahaaWcbeqaaiabdMeajbaakiabgkHiTiabigdaXiabc6caUaaa@4374@

An exhaustive examination of all clusters in a problem of this type is computationally unmanageable for all but a small number of vertices. For example, a 40 vertex graph where all vertices are treated as adjacent to all other vertices has over one-thousand billion potential clusters. Normally, first-order connectivity (where only immediate neighbours are considered adjacent) is used to both limit the size of the cluster search problem, in addition to increasing the usefulness of search results. When a search employs a first-order connectivity constraint, the resulting clusters are connected in the sense that it would be possible to 'walk' from any vertex to any other vertex along the edges of the graph. In this form, it is usually difficult to estimate the exact number of potential clusters searched because complexity is defined by the topology of the graph. Nonetheless, the search process is more manageable, and more importantly, has more epidemiological meaning; 'clusters' of disease imply a process of diffusion, local hazard or common cause, all of which seem more sensible when potential clusters are contiguous systems.

Tango and Takahashi recommend a full enumeration of all possible first-order connected clusters up to a specified size (e.g., a certain number of the nearest neighbours) [7]. This size limit bounds the computational complexity, but also limits the search to clusters that are relatively small. In this form, the value of *w*_*ij *_is the same for all edges in the graph (by simply defining adjacency), and not immediately relevant to how the search proceeds. Although the size limit prevents this strategy from finding large clusters, there are a finite and estimable number of potential clusters within this size constraint. Once a set of potential clusters is found for a given study area, this same set can be used in future searches for clusters in the same study area, thereby saving computing time. It may be possible to spatially index all potential clusters, which could also save time in the Monte Carlo sampling process.

Patil and Taille describe an adjacency constrained approach that restricts the search process to vertices within what they refer to as 'upper level sets' [5,10]. Assunção et al. define this strategy more generally, and refer to it as a 'static minimum spanning tree' approach [8]. In this form, the *w*_*ij *_measures the difference between the disease rates among neighbouring vertices. Minimum spanning trees are grown in a way that minimizes the sum of edge weights, which in this case, is the difference in rates between vertices within the tree. Each stage of tree growth represents a new potential cluster. Unlike the fully enumerative approach, the set of potential clusters is not fixed, and must be recalculated with each new set of case and/or population data. This is because the values of *y*_*i *_and *w*_*ij *_are not constant with different case data (including simulated data). Though the search strategy is relatively efficient, this can add a considerable burden to the Monte Carlo simulations used for hypothesis testing.

The Duczmal and Assunção approach begins by finding a most-likely circular cluster of disease [6]. This is a starting potential cluster set *A*; the complement of this potential cluster set is A¯
 MathType@MTEF@5@5@+=feaafiart1ev1aaatCvAUfKttLearuWrP9MDH5MBPbIqV92AaeXatLxBI9gBaebbnrfifHhDYfgasaacH8akY=wiFfYdH8Gipec8Eeeu0xXdbba9frFj0=OqFfea0dXdd9vqai=hGuQ8kuc9pgc9s8qqaq=dirpe0xb9q8qiLsFr0=vr0=vr0dc8meaabaqaciaacaGaaeqabaqabeGadaaakeaadaqdaaqaaiabdgeabbaaaaa@2DC8@. Any vertex in A¯
 MathType@MTEF@5@5@+=feaafiart1ev1aaatCvAUfKttLearuWrP9MDH5MBPbIqV92AaeXatLxBI9gBaebbnrfifHhDYfgasaacH8akY=wiFfYdH8Gipec8Eeeu0xXdbba9frFj0=OqFfea0dXdd9vqai=hGuQ8kuc9pgc9s8qqaq=dirpe0xb9q8qiLsFr0=vr0=vr0dc8meaabaqaciaacaGaaeqabaqabeGadaaakeaadaqdaaqaaiabdgeabbaaaaa@2DC8@ that is adjacent to any vertex in *A *may be moved from A¯
 MathType@MTEF@5@5@+=feaafiart1ev1aaatCvAUfKttLearuWrP9MDH5MBPbIqV92AaeXatLxBI9gBaebbnrfifHhDYfgasaacH8akY=wiFfYdH8Gipec8Eeeu0xXdbba9frFj0=OqFfea0dXdd9vqai=hGuQ8kuc9pgc9s8qqaq=dirpe0xb9q8qiLsFr0=vr0=vr0dc8meaabaqaciaacaGaaeqabaqabeGadaaakeaadaqdaaqaaiabdgeabbaaaaa@2DC8@ into *A*; likewise, any vertex in *A *can be moved from *A *into A¯
 MathType@MTEF@5@5@+=feaafiart1ev1aaatCvAUfKttLearuWrP9MDH5MBPbIqV92AaeXatLxBI9gBaebbnrfifHhDYfgasaacH8akY=wiFfYdH8Gipec8Eeeu0xXdbba9frFj0=OqFfea0dXdd9vqai=hGuQ8kuc9pgc9s8qqaq=dirpe0xb9q8qiLsFr0=vr0=vr0dc8meaabaqaciaacaGaaeqabaqabeGadaaakeaadaqdaaqaaiabdgeabbaaaaa@2DC8@ provided that this move does not make *A *an discontiguous cluster. Each feasible move is associated with a change in the global objective function – the LR associated with *A*. Moves that increase the global objective function are usually preferred over moves that do not, although a simulated annealing heuristic is incorporated into the decision strategy to ensure the problem is not confined to local optima. This strategy enables the possibility of short-term 'bad' moves to prevent the search process from getting stuck in local optima. Unlike the other methods discussed here, the method does not aim to generate a large number of potential clusters, rather, the search process aims to actively search for a single most-likely cluster by maximizing the objective (the LR associated with *A*). When the search process is finished, *A *is treated as the most-likely cluster, and evaluated using Monte Carlo methods.

As an alternative, Assunção et al. simplify the above strategy into a greedy search from all vertices, rather than a probabilistic search starting from an identified circular cluster [8]. This algorithm, which we refer to as a 'greedy growth search' (GGS), is essentially a greedy hybrid of the Duczmal and Assunção and Tango and Takahashi approaches. The method starts with any *v*_*i*_, which is treated as a potential cluster. Of all *v*_*j *_adjacent to all *v*_*i*_, identify the v_*j *_that results in the largest LR if it were joined with *v*_*i *_to form a new potential cluster. This v_*j *_is added to the potential cluster, and other vertices are added in this same manner until all vertices have been added (or a population or other threshold is met). This process is repeated for all *v*_*i*_. The potential cluster with the largest LR is treated as the most-likely cluster of disease.

The above adjacency constrained disease cluster searches are all able to locate irregularly shaped clusters relatively efficiently. However, evidence suggests that these approaches may have lower power to detect clusters and poorer accuracy in defining cluster shapes than methods that search a specific geometry, such as circles or ellipses (Duczmal, Kulldorff and Huang 2006). A number of strategies are available to offset these challenges. In the remainder of this article, we describe our implementation of the GGS method in detail, and discuss two simple adaptations of the GGS that improve its ability to avoid some of the pitfalls typical of adjacency constrained searches for disease clusters. We then observe the performance of these adaptations on simple simulated data sets.

## Results

### The Greedy Growth Search (GGS)

Here we describe the general procedure that creates the sets of vertices that are evaluated as potential clusters for the GGS approach. Suppose an area is divided into *I *geographic regions: *v*_1_, *v*_2_,...,*v*_*I*_. As above, we treat these regions as vertices on a graph in which edges represent adjacency. Suppose *Z *is a set of connected vertices and *n*_*Z *_is the total number of cases within *Z*. A sequence of sets is constructed for each vertex *v*_*i*_, *i *= 1,...,*I*. For region *v*_*i *_we start with the set *A*_*i*0 _= {*v*_*i*_}, *i *= 1,...,*I*, and define its complement as A¯
 MathType@MTEF@5@5@+=feaafiart1ev1aaatCvAUfKttLearuWrP9MDH5MBPbIqV92AaeXatLxBI9gBaebbnrfifHhDYfgasaacH8akY=wiFfYdH8Gipec8Eeeu0xXdbba9frFj0=OqFfea0dXdd9vqai=hGuQ8kuc9pgc9s8qqaq=dirpe0xb9q8qiLsFr0=vr0=vr0dc8meaabaqaciaacaGaaeqabaqabeGadaaakeaadaqdaaqaaiabdgeabbaaaaa@2DC8@_*i*0 _= *V*\*A*_*i*0_, *i *= 1,...,*I*. We expand on this set by adding one vertex at a time. At the *m*-th step, *A*_*i,m *_= *A*_*i,m*-1 _∪ v*_m _where v*_m _is the region in A¯
 MathType@MTEF@5@5@+=feaafiart1ev1aaatCvAUfKttLearuWrP9MDH5MBPbIqV92AaeXatLxBI9gBaebbnrfifHhDYfgasaacH8akY=wiFfYdH8Gipec8Eeeu0xXdbba9frFj0=OqFfea0dXdd9vqai=hGuQ8kuc9pgc9s8qqaq=dirpe0xb9q8qiLsFr0=vr0=vr0dc8meaabaqaciaacaGaaeqabaqabeGadaaakeaadaqdaaqaaiabdgeabbaaaaa@2DC8@_*i,m*-1_(=*V*\*A*_*i,m*-1_) that is connected to at least one vertex in *A*_*i,m*-1 _and that results in an LR that is larger than any the LR resulting from the addition of any other feasible vertex. More precisely, let B¯
 MathType@MTEF@5@5@+=feaafiart1ev1aaatCvAUfKttLearuWrP9MDH5MBPbIqV92AaeXatLxBI9gBaebbnrfifHhDYfgasaacH8akY=wiFfYdH8Gipec8Eeeu0xXdbba9frFj0=OqFfea0dXdd9vqai=hGuQ8kuc9pgc9s8qqaq=dirpe0xb9q8qiLsFr0=vr0=vr0dc8meaabaqaciaacaGaaeqabaqabeGadaaakeaadaqdaaqaaiabdkeacbaaaaa@2DCA@_*i,m*-1 _be the subset of regions A¯
 MathType@MTEF@5@5@+=feaafiart1ev1aaatCvAUfKttLearuWrP9MDH5MBPbIqV92AaeXatLxBI9gBaebbnrfifHhDYfgasaacH8akY=wiFfYdH8Gipec8Eeeu0xXdbba9frFj0=OqFfea0dXdd9vqai=hGuQ8kuc9pgc9s8qqaq=dirpe0xb9q8qiLsFr0=vr0=vr0dc8meaabaqaciaacaGaaeqabaqabeGadaaakeaadaqdaaqaaiabdgeabbaaaaa@2DC8@_*i,m*-1 _that are connected to a region in *A*_*i,m*-1_, B¯
 MathType@MTEF@5@5@+=feaafiart1ev1aaatCvAUfKttLearuWrP9MDH5MBPbIqV92AaeXatLxBI9gBaebbnrfifHhDYfgasaacH8akY=wiFfYdH8Gipec8Eeeu0xXdbba9frFj0=OqFfea0dXdd9vqai=hGuQ8kuc9pgc9s8qqaq=dirpe0xb9q8qiLsFr0=vr0=vr0dc8meaabaqaciaacaGaaeqabaqabeGadaaakeaadaqdaaqaaiabdkeacbaaaaa@2DCA@_*i,m*-1 _⊆ A¯
 MathType@MTEF@5@5@+=feaafiart1ev1aaatCvAUfKttLearuWrP9MDH5MBPbIqV92AaeXatLxBI9gBaebbnrfifHhDYfgasaacH8akY=wiFfYdH8Gipec8Eeeu0xXdbba9frFj0=OqFfea0dXdd9vqai=hGuQ8kuc9pgc9s8qqaq=dirpe0xb9q8qiLsFr0=vr0=vr0dc8meaabaqaciaacaGaaeqabaqabeGadaaakeaadaqdaaqaaiabdgeabbaaaaa@2DC8@_*i,m*-1_. The next vertex to be added to *A*_*i,m*-1 _is defined as

v*m=arg⁡max⁡x∈B¯i,m−1LR(Ai,m−1∪x).
 MathType@MTEF@5@5@+=feaafiart1ev1aaatCvAUfKttLearuWrP9MDH5MBPbIqV92AaeXatLxBI9gBaebbnrfifHhDYfgasaacH8akY=wiFfYdH8Gipec8Eeeu0xXdbba9frFj0=OqFfea0dXdd9vqai=hGuQ8kuc9pgc9s8qqaq=dirpe0xb9q8qiLsFr0=vr0=vr0dc8meaabaqaciaacaGaaeqabaqabeGadaaakeaacqWG2bGDcqGGQaGkdaWgaaWcbaGaemyBa0gabeaakiabg2da9maaxababaGagiyyaeMaeiOCaiNaei4zaCMagiyBa0MaeiyyaeMaeiiEaGhaleaacqWG4baEcqGHiiIZdaqdaaqaaiabdkeacbaadaWgaaadbaGaemyAaKMaeiilaWIaemyBa0MaeyOeI0IaeGymaedabeaaaSqabaGccqWGmbatcqWGsbGucqGGOaakcqWGbbqqdaWgaaWcbaGaemyAaKMaeiilaWIaemyBa0MaeyOeI0IaeGymaedabeaakiabgQIiilabdIha4jabcMcaPiabc6caUaaa@529A@

The addition of regions continues until the addition of another region would exceed a pre-specified threshold, typically half of the population. Suppose for region *v*_*i*_, that at most *M*_*i *_regions are added to meet this criterion, then ∑i=1IdiI[vi∈Ai,Mi]<12d+≤∑i=1IdiI[vi∈Ai,Mi+1]
 MathType@MTEF@5@5@+=feaafiart1ev1aaatCvAUfKttLearuWrP9MDH5MBPbIqV92AaeXatLxBI9gBaebbnrfifHhDYfgasaacH8akY=wiFfYdH8Gipec8Eeeu0xXdbba9frFj0=OqFfea0dXdd9vqai=hGuQ8kuc9pgc9s8qqaq=dirpe0xb9q8qiLsFr0=vr0=vr0dc8meaabaqaciaacaGaaeqabaqabeGadaaakeaadaaeWaqaaiabdsgaKnaaBaaaleaacqWGPbqAaeqaaaqaaiabdMgaPjabg2da9iabigdaXaqaaiabdMeajbqdcqGHris5aOGaemysaK0aaSbaaSqaamaadmaabaGaemODay3aaSbaaWqaaiabdMgaPbqabaWccqGHiiIZcqWGbbqqdaWgaaadbaGaemyAaKgabeaaliabcYcaSmaaBaaameaacqWGnbqtdaWgaaqaaiabdMgaPbqabaaabeaaaSGaay5waiaaw2faaaqabaGccqGH8aapdaWcbaWcbaGaeGymaedabaGaeGOmaidaaOGaemizaq2aaSbaaSqaaiabgUcaRaqabaGccqGHKjYOdaaeWaqaaiabdsgaKnaaBaaaleaacqWGPbqAaeqaaaqaaiabdMgaPjabg2da9iabigdaXaqaaiabdMeajbqdcqGHris5aOGaemysaK0aaSbaaSqaamaadmaabaGaemODay3aaSbaaWqaaiabdMgaPbqabaWccqGHiiIZcqWGbbqqdaWgaaadbaGaemyAaKgabeaaliabcYcaSmaaBaaameaacqWGnbqtdaWgaaqaaiabdMgaPjabgUcaRiabigdaXaqabaaabeaaaSGaay5waiaaw2faaaqabaaaaa@6470@, where 1 ≤ *M*_*i *_≤ *I*. Thus for region *v*_*i*_, the collection of potential clusters is **A**_*i *_= {*A*_*i*0_, *A*_*i*1_,...,AiMi
 MathType@MTEF@5@5@+=feaafiart1ev1aaatCvAUfKttLearuWrP9MDH5MBPbIqV92AaeXatLxBI9gBaebbnrfifHhDYfgasaacH8akY=wiFfYdH8Gipec8Eeeu0xXdbba9frFj0=OqFfea0dXdd9vqai=hGuQ8kuc9pgc9s8qqaq=dirpe0xb9q8qiLsFr0=vr0=vr0dc8meaabaqaciaacaGaaeqabaqabeGadaaakeaacqWGbbqqdaWgaaWcbaGaemyAaKMaemyta00aaSbaaWqaaiabdMgaPbqabaaaleqaaaaa@31F4@} and the collection of all potential clusters is defined as

**A **= {*A*_1_,...,*A*_*I*_}.

The most-likely cluster is the set in **A **that has the highest associated LR, arg max_*C*∈**A **_*LR*(*C*), and this cluster is tested for statistical significance using the Monte Carlo approach similar to the spatial scan.

In order to avoid some of the problems related to the GGS approach (notably, its tendency to over-fit data with oddly shaped clusters), we describe two adaptations to the approach. One is a penalty applied to the test statistic, and the other affects how the search for clusters terminates.

### Adaptation 1: non-connectivity penalty

Duczmal, Kulldorff and Huang recommend a non-compactness penalty be used to limit the irregular shapes often found when using adjacency constrained searches [9]. This penalty is based on the ratio of a potential cluster's area and the area of a convex hull that encloses the vertices of this potential cluster. This penalty encourages clusters to take a roughly compact form, since the effect of the penalty increases proportionally to a drop in the ratio of a potential cluster's area and the area of a convex hull enclosing it. The magnitude of the penalty can be varied according to prior information about a cluster's shape, the rarity of disease, or the amount of spatial variation in disease rates. The penalty is defined as

K(Z)=B(Z)π(H(Z)2π)2
 MathType@MTEF@5@5@+=feaafiart1ev1aaatCvAUfKttLearuWrP9MDH5MBPbIqV92AaeXatLxBI9gBaebbnrfifHhDYfgasaacH8akY=wiFfYdH8Gipec8Eeeu0xXdbba9frFj0=OqFfea0dXdd9vqai=hGuQ8kuc9pgc9s8qqaq=dirpe0xb9q8qiLsFr0=vr0=vr0dc8meaabaqaciaacaGaaeqabaqabeGadaaakeaacqWGlbWscqGGOaakcqWGAbGwcqGGPaqkcqGH9aqpdaWcaaqaaiabdkeacjabcIcaOiabdQfaAjabcMcaPaqaaGGaciab=b8aWnaabmaabaWaaSaaaeaacqWGibascqGGOaakcqWGAbGwcqGGPaqkaeaacqaIYaGmcqWFapaCaaaacaGLOaGaayzkaaWaaWbaaSqabeaacqaIYaGmaaaaaaaa@40FA@

where *B*(*Z*) is the area of the potential cluster Z and *H(Z) *is the perimeter of a convex hull of the potential cluster Z. The penalized likelihood ratio, PLR(*Z*), generalized to LR(Z)K(Z)α
 MathType@MTEF@5@5@+=feaafiart1ev1aaatCvAUfKttLearuWrP9MDH5MBPbIqV92AaeXatLxBI9gBaebbnrfifHhDYfgasaacH8akY=wiFfYdH8Gipec8Eeeu0xXdbba9frFj0=OqFfea0dXdd9vqai=hGuQ8kuc9pgc9s8qqaq=dirpe0xb9q8qiLsFr0=vr0=vr0dc8meaabaqaciaacaGaaeqabaqabeGadaaakeaacqqGmbatcqqGsbGucqqGOaakcqqGAbGwcqqGPaqkdaahaaWcbeqaaiabbUealjabbIcaOiabbQfaAjabbMcaPmaaCaaameqabaacciGae8xSdegaaaaaaaa@37EA@ is used in place of LR, where *α *usually takes on values between 0 and 1.

As an alternative, we offer a non-connectivity penalty. This method penalizes potential clusters in proportion to the ratio of edges in a potential cluster *Z *to the total possible number of edges in a potential cluster *Z*. Let *e*(*Z*) be the number of edges that connect the number of vertices in potential cluster *Z*. The likelihood ratio statistic becomes

PLR2(Z,α)=LR(Z)K(Z)α
 MathType@MTEF@5@5@+=feaafiart1ev1aaatCvAUfKttLearuWrP9MDH5MBPbIqV92AaeXatLxBI9gBaebbnrfifHhDYfgasaacH8akY=wiFfYdH8Gipec8Eeeu0xXdbba9frFj0=OqFfea0dXdd9vqai=hGuQ8kuc9pgc9s8qqaq=dirpe0xb9q8qiLsFr0=vr0=vr0dc8meaabaqaciaacaGaaeqabaqabeGadaaakeaacqWGqbaucqWGmbatcqWGsbGudaWgaaWcbaGaeGOmaidabeaakiabcIcaOiabdQfaAjabcYcaSGGaciab=f7aHjabcMcaPiabg2da9iabdYeamjabdkfasjabcIcaOiabdQfaAjabcMcaPmaaCaaaleqabaGaem4saSKaeiikaGIaemOwaOLaeiykaKYaaWbaaWqabeaacqWFXoqyaaaaaaaa@4306@

where

K(Z)=e(Z)3(v(Z)−2)).
 MathType@MTEF@5@5@+=feaafiart1ev1aaatCvAUfKttLearuWrP9MDH5MBPbIqV92AaeXatLxBI9gBaebbnrfifHhDYfgasaacH8akY=wiFfYdH8Gipec8Eeeu0xXdbba9frFj0=OqFfea0dXdd9vqai=hGuQ8kuc9pgc9s8qqaq=dirpe0xb9q8qiLsFr0=vr0=vr0dc8meaabaqaciaacaGaaeqabaqabeGadaaakeaacqWGlbWscqGGOaakcqWGAbGwcqGGPaqkcqGH9aqpdaWcaaqaaiabdwgaLjabcIcaOiabdQfaAjabcMcaPaqaaiabiodaZiabcIcaOiabdAha2jabcIcaOiabdQfaAjabcMcaPiabgkHiTiabikdaYiabcMcaPiabcMcaPaaacqGGUaGlaaa@40B9@

*K*(Z) is the ratio of the number of edges in a potential cluster to the total possible number of edges in a potential cluster, which is can be determined based on the number of vertices (*v*(*Z*)) in a potential cluster set. As with the previous penalty function, *α *is a user specified scaling value. The non-connectivity penalty is applied in the same manner as the non-compactness penalty. As values of *α *approach 0, the penalty has little effect on LR; as values increase above 1, the effect of the penalty increases. As the penalty increases, clusters tend to take on forms in which there are more connections between vertices, which generally results in more compact shapes.

**Figure 2:**
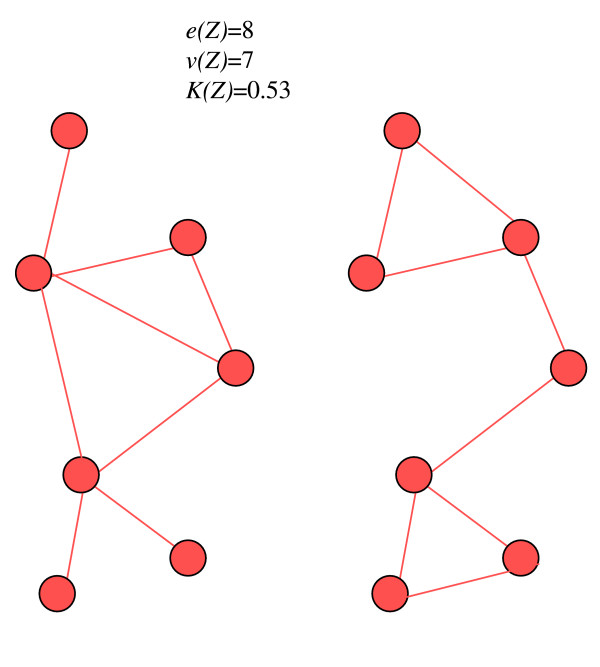
Two irregular clusters with the same non-connectivity penalty.

The rationale for the non-connectivity penalty is similar to the non-compactness penalty, with the key conceptual difference that the former is not explicit about shape, merely about the structure of adjacency. In this respect, there can be considerable variability in the shape that two potential clusters with the same non-connectivity penalty may take (Figure 2). This may be an advantage when a more relaxed penalty is required – such as when clusters are small and suspected to be irregular, or when the disease is common and highly irregular cluster shapes are of less concern.

### Adaptation 2: depth limit

**Figure 3:**
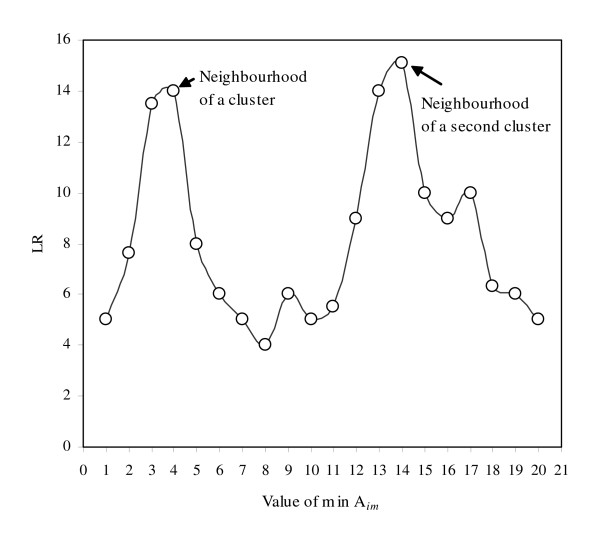
Illustration of how a search proceeds within a spatial neighbourhood.

In the GGS approach, as a search from a particular vertex proceeds, the LR associated with the new potential cluster changes depending on the neighbouring disease rate. When a search is in the general geographical area (or 'neighbourhood') of vertices with high disease rates, LRs associated with potential clusters are larger than when a search is in the neighbourhood of vertices with low disease rates. This results in a search profile, associated with each search from *v*_*i*_, that varies depending on the disease characteristics of the neighbourhood in which the search is taking place (Figure 3). When the objective is to find high local disease clusters, we suggest that it may be worthwhile to stop a search that has descended into a trough, or low point, even if it were to climb out of the trough if more vertices were added. We apply a 'depth limit' to control the length of time a search sequence will stay within a trough. The GGS search proceeds in the normal manner; for each search from *v*_*i*_, a feasible node *v*_*j *_is added that results in the largest LR. If the LR associated with a search from a particular vertex has not increased to a new high-point in the search after a specific number of moves (the depth limit), the search stops, and moves to a new *v*_*i*_.

In the framework of the GGS method above, the stopping point *M*_*i *_for the sets associated with region *v*_*i *_is not necessarily based on a proportion of the population in the potential cluster search process. Instead, failure to increase the LR to a new high-point after *u *steps (*1 *<*u *<*I*) stops the creation of sets for region *v*_*i *_and *M*_*i *_is the last of those *u *steps. In particular, *LR*(*A*_*i,Mi-u*_) > *LR*(*A*_*i,Mi-u+1*_),...,*LR*(*A*_*i,Mi-u*_) > LR(*A*_*i,Mi*_). The parameter *u *is set by the user, and takes on values from 0 to *I*. Large values of *u *relaxes the search constraint (allowing searches to descend into troughs) and a value of 0 means that searches are terminated if no vertices can be added that increase the LR.

Terminating searches with the depth limit helps prevent distinct clusters from being combined into one super-set composed of several clusters. This may be particularly important when there is a meaningful physical separation between real clusters of disease – perhaps because the forces influencing these clusters differ from place to place. In Figure 3, we can see how the LR might change while searching in the neighbourhood of two geographically distinct clusters. The largest LR is found when the clusters are combined, but arguably, these clusters should be thought of as distinct, since there is a large trough in the LR separating them. The depth limit can be used to better define the distinct geographies of multiple clusters. An added benefit of the depth limit is its ability to speed up the search process; the number of sets created in a search spawned from *v*_*i *_is smaller when a depth limit is applied than when no depth limit were applied.

### Experiments

We design a simple experiment based on synthesized data in order to evaluate the effectiveness of these two adaptations of the GGS. The emphasis of this experiment is on the ability of these adaptations to define the boundaries of clusters of regular (circular) and irregular shape. The experiment uses simulated data. These simulated data are a simplified representation of disease patterns in the real world, but give a suitable baseline for understanding the general behaviour of these two adaptations. The algorithms for solving and testing the GGS method and its two adaptations were programmed in the SAS^® ^language [11].

Our experimental study space consists of an approximately square tessellation of 203 hexagons (or 'regions). The geometric centres of the regions are the vertex set, and the edge set is defined by immediate neighbours – hexagons that share line segments. In all experiments, each region receives an equal population of 'persons', and the study space is divided into two sets – a cluster area set and a baseline area set. For each person in each region, we determine whether or not they are a case based on the level of risk associated with the set in which their resident region falls. Regions inside synthesized clusters have higher levels of risk than regions outside the synthesized clusters.

**Figure 4:**
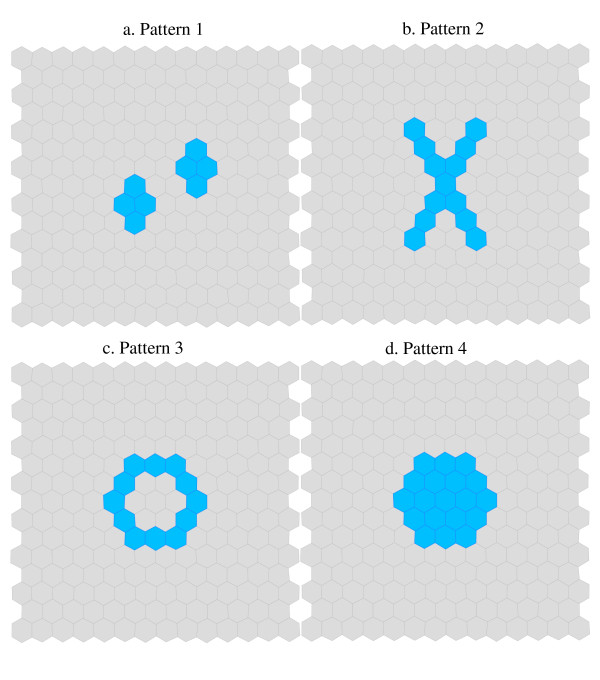
The four cluster patterns.

We base our experiment on 16 different scenarios. The scenarios consist of four different clusters area set patterns (two small clusters, an 'X' shape, a 'ring' shape and a 'large circular' shape (Figure 4)) two region population levels (1,000 persons per region and 10,000 persons per region) and two pairs of disease rates associated with the cluster area and baseline area. We chose these population sizes because they reflect the population characteristics common to Census areas in Canada and elsewhere – such as census tracts and census blocks. Although our experiment assumes an homogenously distributed population and is therefore likely to under-represent the variability in disease incidence likely in the real world, we prefer the homogenous distribution to simplify the interpretation of results. We acknowledge, however, that the results of our experiments are likely to be less variable than would be observed in real world situations. In all scenarios, the baseline rate is 2.5 per 1,000. For the ring, circle and 'X' cluster scenarios, the cluster rates are 5 per 1,000 and 7.5 per 1,000. For the two circle cluster scenarios, there is a primary cluster with same cluster rate as above, and a secondary cluster that is half-way between the cluster rate and the baseline rate (3.75 per 1,000 and 5 per 1,000).

The magnitudes of the non-connectivity penalty (defined in terms of *α*) and depth limit (defined in terms of *u*) are varied systematically in order to observe how the adaptations affect the method's ability to find clusters. For each scenario, we vary the combination of settings *α *and *u *from 0 to 4 in 1 unit increments, resulting in 25 different combinations. In total, there are 16 different scenarios, and each scenario is associated with 25 different *α *and *u *setting combinations.

For each scenario, the GGS is used to search for a most-likely cluster, with the settings of *α *and *u *as described above. We measure success in terms of true positives (regions correctly identified as existing inside a synthesized cluster) and false positives (regions incorrectly identified as existing inside a synthesized cluster) for each scenario. Each scenario is repeated 100 times, and the grand sum of true positives and false positives are recorded for each scenario; that is, the sum of true positives and false positives found for a given scenario over 100 repetitions. We calculate measures of sensitivity (grand total of found true positives/grand total of actual true positives) and positive predicted value (grand total of found true positives/the sum of the grand total of found true and found false positives). The values of sensitivity and positive predicted value vary from 0 to 1, and larger values are preferred over smaller values.

### Findings from the simulations

We present the tabulated results of the scenarios in Additional files 1 and 2. The results described in detail below are based on data from Additional file 1, in which the difference between the cluster rates and baseline rates is smallest.

**Figure 5:**
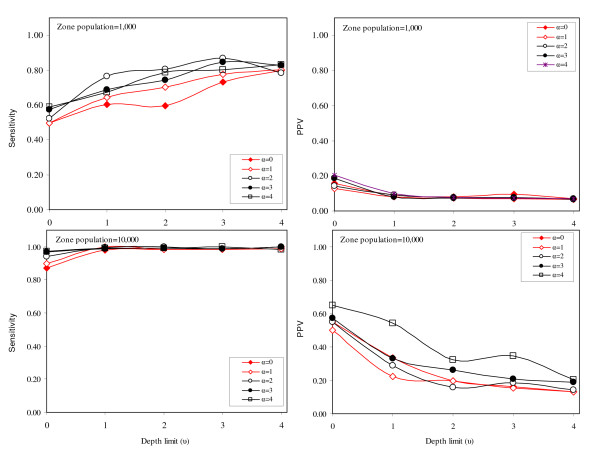
Sensitivity and positive predicted value for cluster pattern 1 ('two clusters').

For the 'two cluster' pattern we synthesize a main cluster and a secondary cluster. For this experiment, we assume that the objective of the cluster detection process is to find the main cluster. The purpose of synthesizing a secondary cluster is to observe the effect of a more complex spatial landscape on the two adaptations of the GGS. Sensitivity and positive predicted value (PPV) do not appear to differ greatly for the different adaptation settings (Figures 5). For scenarios based on smaller region populations (n = 1,000), larger values of *u *and *α *correspond with a higher sensitivity. PPV, on the other hand, is uniformly low. For scenarios based on larger region populations (n = 10,000), sensitivity is high for all values of *α *and *u*. PPV is highest when the *u *is small (i.e., the depth limit is shallow).

**Figure 6:**
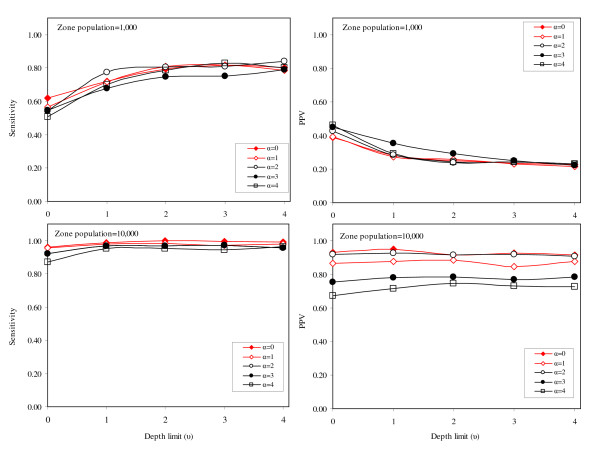
Sensitivity and positive predicted value for cluster pattern 2 ('X').

**Figure 7:**
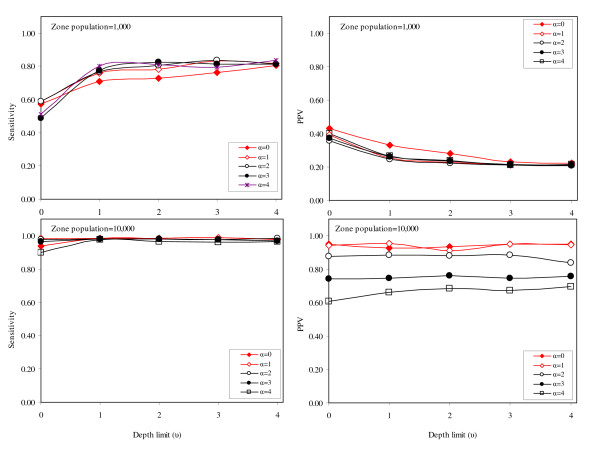
Sensitivity and positive predicted value for cluster pattern 3 ('Ring').

**Figure 8:**
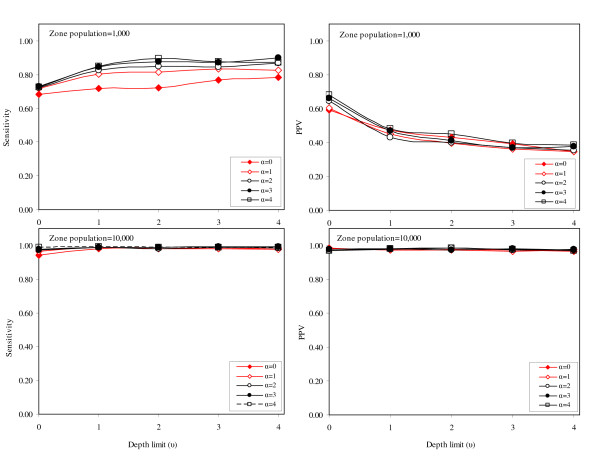
Sensitivity and positive predicted value for cluster pattern 4 ('Circle').

For the 'X' cluster pattern, sensitivity and PPV are similar for all scenarios based on smaller region populations (Figure 6). The shape and magnitude of PPV and sensitivity curves do not differ greatly, particularly when values of *α *and *u *are greater than zero. For scenarios with the larger region populations, sensitivity is similar for all values of *α *and *u*, though marginally higher for the lower values of *α*. However, PPV varies considerably depending on the value of *α*; as *α *increases (and the non-connectivity further penalizes potential clusters of irregular shape) the PPV drops. For the 'ring' cluster pattern (Figure 7), the patterns of PPV and sensitivity are very similar to the observations for the 'X' cluster pattern. For small region populations, the PPV and sensitivity vary little with the changing values of *α *and *u*, but when the region population is large, PPV is highest when the non-connectivity penalty is relaxed (*α *= 0). For cluster pattern 4 (where the synthesized cluster takes the shape of a large central circle), the PPV and sensitivity graphs are similar in shape and magnitude to the graphs associated with cluster pattern 1 (Figure 8). For smaller region populations, the PPV is similar across all values of *α *and *u*, however, larger values of *α *are generally better. For larger region populations, PPV and sensitivity are very high, and do not vary noticeably for different levels of *α *and *u*.

**Figure 9:**
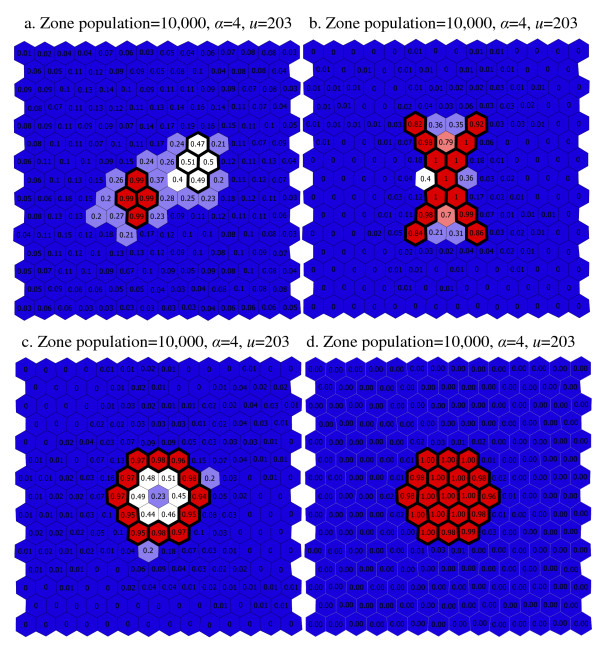
a–d Maps of scenario results without the use of a depth limit.

To help visualize the changes in sensitivity and PPV as a function of the different settings of *α *and *u*, we show maps of the simulated results found in Appendix 1. The values associated with the regions are the proportions of the simulations (for a given scenario) that a region is identified as part of a found cluster. For regions inside a cluster area, high values are desirable. For regions outside a cluster area, low values are desirable. Figure 9a–9d show the maps where *α *= 4 and *u *= 203 (that is, where there a relatively high non-connectivity penalty and no depth limit). In the scenarios used to generate these maps, regions have populations of 10,000. For all but 'large circle' cluster pattern, a number of regions are frequently and incorrectly identified as part of a synthesized cluster area, though the inaccuracy is confined to the regions adjacent to the synthesized clusters. For the 'large circle' pattern the detected clusters are confined to the synthesized cluster area.

**Figure 10:**
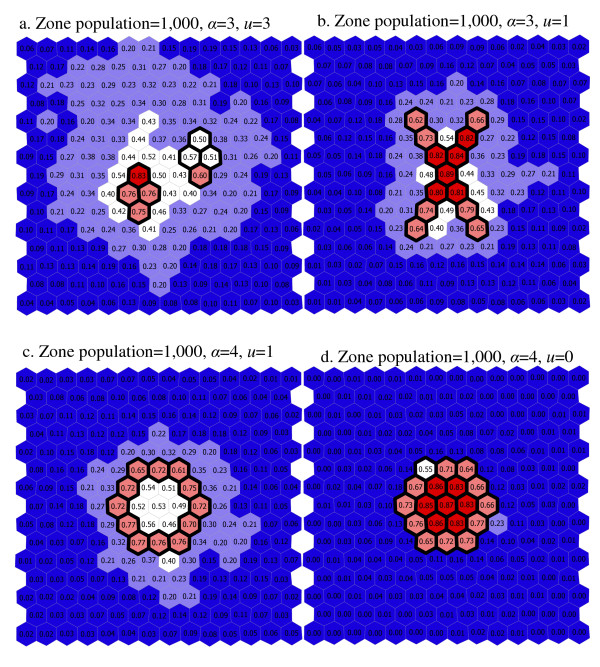
a–d. Maps of scenario results with the best values of *α *and *u *(region populations of 1,000).

**Figure 11:**
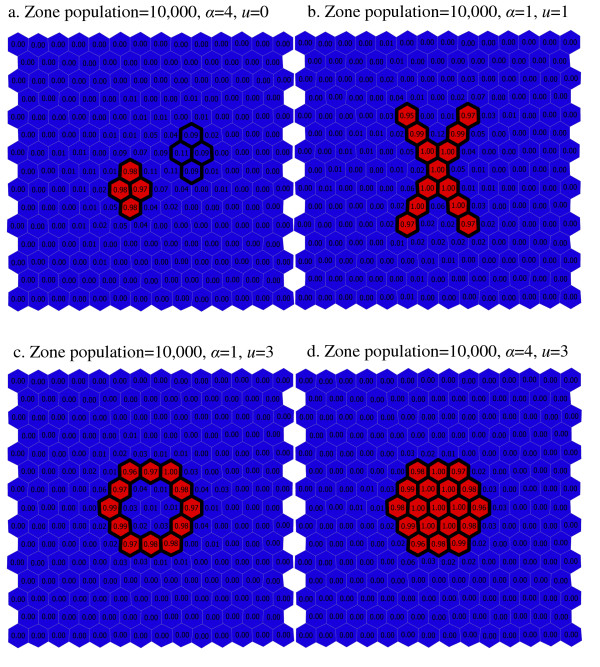
a–d. Maps of scenario results with the best values of *α *and *u *(region populations of 10,000).

In figures 10a–10d and 11a–11d, each map is based on the settings of *α *and *u *that produced the highest combination of sensitivity and PPV for a given synthesized cluster and a given region population size. For the scenarios in which region populations are small (10a–10d), the number of falsely included regions is high for all but the 'large circle' cluster pattern. Many of the falsely detected regions are near the synthesized cluster shapes, but these maps also indicate that found clusters are frequently quite large, and irregularly shaped. For the high region population scenarios (11a–11d), accuracy is higher. Most regions are correctly identified, and few regions are incorrectly identified. For the two cluster scenario (11a) the found clusters are confined to the higher of the two synthesized cluster areas.

## Discussion

The evidence from our simulations suggests that the non-connectivity penalty and the depth limit can both improve the accuracy with which shapes of clusters are detected, and the mixture of these two adaptations may help identify the correct shape and location of disease clusters. As has been observed with a non-compactness penalty (Duczmal, Kulldorff and Huang 2006), the non-connectivity penalty prevents the found clusters from taking on unusual, 'octopus-like' shapes even when there is no depth limit. When the true cluster shape is compact, this is a useful constraint on the search process. When the non-connectivity penalty is completely relaxed (*α *= 0), the GGS algorithm is often at its worst, and less sensitive to detecting the correct regions as part of a true cluster. For scenarios in which the cluster pattern is compact (the 'large circle' and 'two cluster' patterns), the strictest non-connectivity penalty (*α *= 4) corresponds to the highest PPV and sensitivity at both population sizes.

When the depth limit is relaxed completely and *u *is equal to the number of regions in the study area (*u *= 203) the PPV and sensitivity are roughly the same as when the depth limit is more restrictive (*u *= 3). This observation is not true when the cluster is irregularly shaped however. For the 'Ring', 'X' and 'two-cluster' patterns, we see a larger proportion of regions falsely included in found clusters when the depth limit is relaxed (*u *= 203) than when a depth limit is applied (as seen in the comparison of 8a–c and 10a–c). These considerations are important since it would appear that using the depth limit may be advantageous when the true cluster shape is irregular, and that its use is not deleterious when the cluster shape is compact. For the 'two-cluster' pattern, the depth limit also helps separate the primary cluster from a nearby secondary cluster. In Figure 8a (*α *= 4, *u *= 203), a number of regions are frequently identified as part of a found most-likely cluster. In 10a (*α *= 4, *u *= 0) the detection process is much more precise, focusing almost entirely on the regions inside the primary cluster.

One of the obvious challenges of using these adaptations in the search for clusters is that they necessitate more prior decision making on the part of the analyst. In order to ensure that the GGS and similar algorithms define clusters precisely, an analyst must specify the correct settings for *α *and *u*. Without already knowing the shape of the cluster ahead of time, this may be a difficult, and at times arbitrary, task. These decisions eliminate much of the elegance of the original spatial scan, and also introduce a possible pre-selection bias. For infectious disease surveillance purposes, these limitations are particularly noteworthy, since a common goal of cluster detection in this setting is to detect the presence of an anomaly or signal, which is then followed up by field investigations and other verification exercises. The reward for finding the precise boundaries of a cluster may not be particularly high. For chronic disease surveillance – where prevalence is relatively high, and reporting times are of courser grain – finding the correct regions may be of more importance. Knowing the precise regions where risk is high helps inform longer-term planning, and provides a more precise indication of where further field investigation or epidemiological study is required.

Fortunately, our results show a degree of consistency across the scenarios, suggesting that there may be some rules of thumb that can guide the use of these two adaptations. For rare events, small population study areas, or for diseases in which there is a low magnitude of variation in disease rates, these (and similar) adaptations are useful for helping the search process to find plausible cluster shapes. In these cases, the exact cluster shape and location may always remain elusive. However, by inhibiting the search process with these constraints, and in particular, the non-connectivity penalty, one is likely to find circular or compact shaped 'hot-spots' that are of most interest in disease surveillance. Although applying a depth limit seems of most value when the true disease cluster is irregularly shaped, or when it is important to keep detected clusters distinct, since it does not appear to inhibit the detection of compact cluster shapes, it may be the preferred adaptation for common diseases, or at lower spatial resolutions. Within the limited scope of our experiment, using both adaptations does not appear to harm the search process most of the time, although using the non-connectivity penalty may prevent the algorithm from finding true irregular patterns when they are present.

In the future, one way of managing these adaptations may be to employ meta-heuristics or cross-validation strategies that search for 'optimal' combinations of the depth limit and non-connectivity penalty for a given problem. For example, one could search for the most suitable values of *u *and *α *on historical data – tuning the parameters based on the LR test statistic, qualitative assessments of patterns, or other evaluative criteria. The settings of *u *and *α *derived from this process could then be used for prospective surveillance or cluster detection purposes. Under these circumstances, the decisions about the role of the two adaptations are informed by historical data, rather than purely arbitrary choices on the part of the analyst.

For exploratory research purposes, experimenting with these adaptations may itself be informative. Observing systematic changes in the shapes of found clusters, while values of *u *and *α *are varied, may suggest the presence of an underlying structure or geography. If, for example, the pattern of found clusters remains compact and circular whatever the values of *u *and *α*, this may indicate the presence of an underlying circular or compact structure. The rationale for this is simple: when a search process is not specific about shape, but a potentially interesting shape is found, we have some (albeit exploratory and un-falsifiable) evidence that the shape might be meaningful. If the pattern of the cluster changes considerably with different values of *u *and *α*, this could suggest that the patterns are more complex, and that a large single geographic structure is absent. In these capacities, the cluster detection process provides exploratory information about what the approximate shape of a cluster may be, in addition to the traditional information about where and what size a cluster is. Though not conclusive, when such findings are consistent with other geographic features of the physical or human landscape, these observations could justify further examination.

In several ways our experimental design may limit the scope of our findings. First, we conducted no power tests. PPV and sensitivity are useful for understanding how the algorithm adaptations are suited to identifying the correct boundaries of a cluster of high risk. Power tests are useful for determining how effective methods are in identifying the presence of statistically noteworthy clusters. The former was our emphasis since adjacency-constrained scan approaches have yet to resolve the best approaches for finding a cluster without including a large number of false-positive regions. As the search methods are refined, power tests will become more important.

Second, we simulated simple disease landscapes with very little non-random variation in risk. Except for the 'two cluster' scenario, all scenarios included a single cluster and non cluster partition of the study area. In the real world, variation is likely to be less discrete, and much more variable. Future work should consider the issue of detecting independent clusters in a landscape of considerable variability. We believe that the depth limit (or a similar adaptation) could be an important tool for work in this area.

Third, the difference in risk between the cluster areas and baseline areas was relatively large (with relative risks either twice or three times higher in the cluster areas). These settings were chosen because they reflect the importance of identifying clinical rather than statistical significance common to chronic disease surveillance, particularly when regional populations are large, or the disease is common. For common diseases, cluster detection methods will often identify clusters of statistical significance but with small effect sizes (for example, with relative risk under 1.2). In this study, we stress the importance of accurately identifying the correct regions within a true cluster of clinical significance, which is likely to be important for more common conditions – where the general presence and significance of a high risk area is likely to be known, but where its precise shape or form may be of independent interest. In rare disease situations, the circular spatial scan may remain a preferred approach, since in these cases the objective is to identify the presence and approximate locations of anomalies and outbreaks, for which this approach remains both a powerful and elegant standard.

## Conclusion

We believe that the two adaptations we propose contribute to the growing toolbox of spatial scan methods for use in disease cluster detection. Methods that can find irregularly shaped clusters are important, but must not sacrifice the simplicity required in surveillance applications. We think that irregular disease cluster detection may be of particular interest for common diseases, or diseases known to be strongly influenced by features of the physical environment – such as waterways, roads or geological structures. In both cases, the use of these and other heuristic adaptations will be critical for ensuring that the found clusters are plausible and meaningful. We see considerable opportunity for use of these methods for exploratory spatial analysis and surveillance of chronic disease and injury. For routine and real-time surveillance of rare disease outbreaks, we would recommend that the non-connectivity penalty is particularly strict so that the adjacency-constrained approaches perform in a manner similar to the traditional spatial scan, or that the traditional spatial scan is used in their place.

## Competing interests

The author(s) declare that they have no competing interests.

## Authors' contributions

NY devised the methods used in the study, wrote the necessary computer programs, conducted the experiments, and wrote the manuscript. RJR participated in the writing of the manuscript, articulation of the methods and the study design. JH aided in the study design and methodology, and assisted in the writing and editing of the manuscript. All authors read and approved the final manuscript.

## Supplementary Material

Additional file 1 Simulation results where cluster area rate is 5 per 1000 and baseline rate is 2.5 per 1000. The data in this table summarize the difference in sensitivity and positive predicted value for the different cluster scenarios with different settings for the non-connectivity penalty and the depth limit. Click here for file

Additional file 2 Simulation results where cluster area rate is 7.5 per 1000 and baseline rate is 2.5 per 1000. The data in this table summarize the difference in sensitivity and positive predicted value for the different cluster scenarios with different settings for the non-connectivity penalty and the depth limit. Click here for file
